# Slightly acidic electrolyzed water inhibits inflammation induced by membrane vesicles of *Staphylococcus aureus*

**DOI:** 10.3389/fmicb.2023.1328055

**Published:** 2024-01-12

**Authors:** Yuko Shimamura, Yukino Oura, Madoka Tsuchiya, Yuka Yamanashi, Asako Ogasawara, Minami Oishi, Misaki Komuro, Kuniaki Sasaki, Shuichi Masuda

**Affiliations:** ^1^School of Food and Nutritional Sciences, University of Shizuoka, Shizuoka, Japan; ^2^Faculty of Science and Engineering, Iwate University, Morioka, Japan

**Keywords:** slightly acidic electrolyzed water, *Staphylococcus aureus*, membrane vesicles, atopic dermatitis, inflammation

## Abstract

*Staphylococcus aureus* grows in the skin of patients with atopic dermatitis and the associated symptoms are induced by membrane vesicles (MVs). This study explored the effects of slightly acidic electrolyzed water (SAEW) on the expression of virulence factors of *S. aureus* and MV-induced inflammation to uncover the potential of SAEW as a new treatment method for atopic dermatitis. Expression levels of genes related to virulence factors in *S. aureus* was assessed and *S. aureus*-derived MVs were characterized. Moreover, expression level of MV-induced Type I allergic reaction-related genes in RBL2H3 cells was also assessed. Significantly decreased staphylococcal enterotoxin A production and decreased virulence factor-related gene expression were observed after culturing *S. aureus* in broth supplemented with SAEW at ratios of 1, 2, and 5 per broth. MVs prepared by culturing *S. aureus* in SAEW-supplemented broth exhibited altered particle size and markedly reduced staphylococcal enterotoxin A content under all addition conditions; moreover, those obtained at a ratio of 1:5 (broth:SAEW) exhibited a reduction in the expression of several proteins associated with hemolytic activity and free iron uptake. The MVs prepared in SAEW-supplemented broth also exhibited remarkably reduced allergy-related gene expression levels in rat cell lines derived from basophilic leukemia-2H3 cells. Overall, SAEW is expected to suppress atopic dermatitis symptoms through the alteration of the properties of *S. aureus*-derived MVs.

## 1 Introduction

The integrity of human skin relies on its microflora to maintain a natural barrier function, effectively preventing harmful bacteria and substances from settling on the skin. Dermal microorganisms cause skin diseases such as atopic dermatitis (AD). AD is a chronic itchy eczema with alternating periods of flares and remissions involving skin barrier function impairment and skin inflammation. Studies have linked *Staphylococcus aureus*, a common human skin bacterium, to AD pathogenesis. For instance, *S. aureus* predominates disease sites in AD-affected individuals in proportion to symptom intensity, with a marked loss in diversity of bacterial flora (Kong et al., 2012). A rapid increase in *S. aureus* population in the epidermis induces AD in mouse models (Kobayashi et al., 2015). Furthermore, membrane vesicles (MVs) released by *S. aureus* can contribute to AD development and exacerbation (Betley and Mekalanos, 1985; Coleman et al., 1989).

Membrane vesicles can transport various substances, including virulence factors, adhesion factors, DNA, RNA, intercellular communication signaling substances, and immunomodulators (O’Regan et al., 2008). *S. aureus*-derived MVs contain not only extracellular and cytoplasmic proteins but also multiple virulence factors (Kobayashi et al., 2015). The presence of β-lactamases in MVs, which confer antibiotic resistance to ampicillin-sensitive gram-negative and gram-positive bacteria in these MVs, suggests their involvement in the spread of drug resistance (Liu et al., 2022). In addition, certain *S. aureus*-derived MVs contain a toxin, which induces cell death by adhering to host cells and delivering the toxin (Jun et al., 2017). Staphylococcal enterotoxin A (SEA), an MV-encapsulated toxin, causes food poisoning and systemic inflammatory reactions, causing AD (Brown et al., 2015). Although MVs function as carriers of toxic factors to hosts and bacteria, *S. aureus*-derived MVs lead to AD induction and exacerbation (Coleman et al., 1989). This highlights the importance of controlling MVs.

Recent studies have revealed the role of skin microflora in AD pathogenesis. In Europe and the United States, “bleach bath therapy,” which involves patients bathing twice a week in a bath containing a certain concentration of sodium hypochlorite used for disinfection, is recommended in AD treatment guidelines as a bacteriostatic treatment against *S. aureus* (Krakowski et al., 2008). Randomized controlled trials on bleach baths for AD revealed a 22% improvement in clinician-reported severity among patients with moderate to severe AD, with 1 in 10 patients experiencing a 50% reduction in severity (Bakaa et al., 2022). However, a meta-analysis of five studies examining bleach bath therapy for AD could not prove its efficacy (Flohr et al., 2014). Administration of bleach bath therapy at the recommended concentrations did not exhibit clear bacteriostatic effects, and part of the observed improvement could be associated with antipruritic function and reinforcement of the skin barrier.

Slightly acidic electrolyzed water (SAEW), a disinfectant solution with a lower available chlorine concentration (ACC) than sodium hypochlorite solution, has a comparable disinfectant effect and is used as a food additive in Japan. SAEW (pH 5.0–6.5) contains hypochlorous acid (Forghani et al., 2015) and is prepared by electrolysis of HCl or an aqueous mixture of HCl and NaOH using an electrolyzer without using a separation membrane between the anode and cathode (Xuan et al., 2017). The ACC of SAEW ranges from 10 to 80 mg/L (Hao et al., 2012), and it possesses bactericidal effect against *S. aureus* and *Listeria monocytogenes* (McCarthy and Burkhardt, 2012; Li et al., 2017), SAEW can be used for AD treatment. However, the effect of SAEW on the toxicity induced by *S. aureus*-derived MVs remains unexplored. Therefore, this study explores the effect of SAEW not only on the growth and expression of virulence factors of *S. aureus* but also on the expression of inflammation induced by *S. aureus*-derived MVs.

## 2 Materials and methods

### 2.1 SAEW

Slightly acidic electrolyzed water was obtained using an SAEW generator (Purester μ-Clean; Morinaga Milk Industry Co., Ltd., Tokyo, Japan). SAEW is a chlorine disinfectant (food additive) with an ACC of 45 ± 2 ppm and pH 5.7. SAEW has been verified as a non-irritating substance that does not cause problems even if it adheres to the skin.

### 2.2 Measurement of viable counts

*Staphylococcus aureus* C-29 (SEA-producing human hand isolate) was used (Shimamura et al., 2006). *S. aureus* C-29 was inoculated into brain heart infusion (BHI) broth and incubated at 37°C for 24 h with shaking (110 rpm). This culture solution (30 μL) was inoculated into 3 mL of BHI broth and incubated at 37°C for 18 h with shaking. *S. aureus* culture solution (10 μL) was inoculated in the BHI:SAEW mixture at different ratios to obtain a total solution volume of 1 mL, and the solution was incubated at 37°C for 24 h with shaking (110 rpm). The mixing ratios of BHI broth:SAEW were 1:1, 1:2, 1:5, 1:6, 1:7, 1:8, 1:9, 1:10, 1:14, and 1:19 (1 mL total volume). Sterile water was used as a control instead of SAEW. After 24 h of incubation, 200 μL of each sample was placed in a flat-bottomed 96-well microplate, and the absorbance at 660 nm (OD_660_) was measured using a plate reader. *S. aureus* was grown in BHI broth and the CFU/mL at OD_660_ was obtained for 0, 3, 4, 5, 6, 7, 8, 9, 10, 17, 20, and 24 h. The following Eq. 1 was obtained, where y represents OD_660_ and x represents the number of bacteria.


(1) y=0.291⁢l⁢n⁢(x)-0.8309

### 2.3 Expression levels of genes related to virulence factors in *S. aureus*

*Staphylococcus aureus* C-29 was inoculated into BHI broth and incubated at 37°C for 24 h with shaking. This culture solution (30 μL) was inoculated into 3 mL BHI broth and incubated at 37°C for 18 h with shaking. Then, 10 μL of the culture solution was added to obtain the BHI broth:SAEW ratios of 1:1, 1:2, and 1:5 (1 mL total volume) and incubated at 37°C for 6 h with shaking (110 rpm). Sterile water was used as a control instead of SAEW. This culture solution was centrifuged (11600 × *g*, 3 min). The supernatant was used to determine SEA production (see below) and the pellet was used to extract RNA. RNA was extracted from the pellet using RiboPureTM-Bacteria (Invitrogen, MA, USA) following manufacturer’s instructions. The purity and concentration of total RNA were measured using a K2800 Nucleic Acid Analyzer (Beijing Kaiao Technology Development Co., Ltd., Beijing, China). cDNA was extracted using PrimeScript RT regent Kit (TaKaRa Bio Inc., Shiga, Japan) and stored at −20°C until use. RT-PCR was performed using Thermal Cycler Dice Real-Time System II (TaKaRa Bio Inc.). The targeted pathogenic factors were SEA (*sea*), RNAIII, β-hemolysin (*hlb*), and the biofilm formation gene (*ica*A). The 16S rRNA gene was used as an internal standard to correct for mRNA levels between samples. Table 1 contains the primer sequences used.

**TABLE 1:** The sequence of primers of genes related to virulence factors in *Staphylococcus aureus*.

Primer	Gene	Sequence (5′ to 3′)
*sea* F	*sea*	GATCAATTTATGGCTAGACG
*sea* R		CGAAGGTTCTGTAGAAGTATGA
RNAIII F	RNAIII	CGATGTTGTTTACGATAGCTT
RNAIII R		CCATCCCAACTTAATAACCA
*ica*A F	*ica*A	AGTTGTCGACGTTGGCTA
*ica*A R		CCAAAGACCTCCCAATGT
*hlb* F	*hlb*	GCGGTTGTGGATTCGATAAT
*hlb* R		CAGCACCACAACGTGAATCT
16S rRNA F	16S rRNA	GCGAAGAACCTTACCAAATC
16S rRNA R		CCAACATCTCACGACACG

### 2.4 Preparation of *S. aureus*-derived MVs

*Staphylococcus aureus* C-29 was inoculated into BHI broth and incubated at 37°C for 24 h with shaking. This culture solution (30 μL) was inoculated into 3 mL of BHI broth and incubated at 37°C for 18 h with shaking. Then, 500 μL of the culture solution was inoculated in BHI:SAEW mixture at different ratios (1:1, 1:2, and 1:5) to obtain a total volume of 200 mL and incubated at 37°C for 20 h with shaking (110 rpm). Sterile water was used as a control instead of SAEW. The resulting culture solution was centrifuged at 3,000 × *g* for 15 min, and the bacterial pellet was removed. The supernatant was filtered through a 0.2-μm filter (Advantec Toyo Co., Ltd., Tokyo, Japan) and further centrifuged at 3,000 × *g* for 30 min using an Amicon Ultra centrifugal filter device 100K (Merck, Darmstadt, Germany). A 100-kDa cutoff (>100 kDa) concentrate was added to the ultracentrifugation tubes (11PA thick-walled tubes, Hitachi Koki Co., Ltd., Tokyo, Japan) and subjected to ultracentrifugation (150,000 × *g*, 4°C, 3 h) using the Himac CP-WX Series separation ultracentrifuge (Hitachi Koki Co., Ltd.). The pellet was resuspended in 50 μL PBS for precipitates obtained from 50 mL of the culture solution, and the amount of protein was measured using a microspectrophotometer (Nikko-Hansen Co., Ltd., Osaka, Japan). The isolated MVs were stored at −20°C until use. Unless otherwise stated, the MVs were used without dilution.

### 2.5 Transmission electron microscopy (TEM) analysis

Each isolated *S. aureus*-derived MV was diluted 10-fold in PBS and subjected to negative staining with 1% aqueous uranyl acetate. The MVs were observed using a JEM-2100 microscope (JEOL, Tokyo, Japan) at an acceleration voltage of 80 kV.

### 2.6 Measurement of the particle size distribution of MVs

Each isolated *S. aureus*-derived MV (30 μL) was diluted with 970 μL of PBS. The particle size distribution of MVs was analyzed using dynamic light scattering (DSL; Zetasizer Ultra ZS Particle Analyzer, Malvern, UK). The particle size was evaluated using the number criterion (Number).

### 2.7 Measurement of SEA in the culture supernatant and MVs

The culture supernatant or each isolated *S. aureus*-derived MV was heated in a sample buffer (1 M Tris–HCl (pH 6.8), 10% SDS, 0.5 mg/mL bromophenol blue, 25% 2-mercaptoethanol, and 20% glycerol) for 5 min. Proteins were separated using SDS-PAGE using 15% polyacrylamide gel. Separated proteins were transferred from the gel to a PVDF membrane using a semidry transfer unit (400 mA, 90 min; Oriental Land Co., Ltd., Tokyo, Japan). After transferring the bands, the PVDF membrane was immersed in a blocking solution (5% non-fat milk powder for blotting; FUJIFILM Wako Pure Chemicals Corporation, Osaka, Japan) for 90 min at room temperature (20°C–25°C). After blocking, PVDF membranes were immersed in a primary antibody solution of anti-rabbit SEA (Sigma-Aldrich, MO, USA), diluted to 1:10,000 (v/v) in PBS, and stored at 4°C overnight. The PVDF membranes were then washed three times with wash solution (20 × Wash Solution Concentrate; KPL, MD, USA) for 5 min, followed by immersion in a secondary antibody dilution of anti-rabbit peroxidase IgG (KPL) diluted 1:1,000 (v/v) in PBS for 90 min at room temperature. The PVDF membranes were washed twice for 5 min in wash solution and once for 5 min in PBS, followed by incubation with the substrate solution (BCIP/NBT, KPL) at room temperature for 30 min. After obtaining the stained images, the membranes were washed with water and air dried and bands were quantified using image analysis software (ImageJ; National Institutes of Health, Bethesda, MA, USA).

### 2.8 SDS-PAGE and LC–MS/MS

The pattern of MV protein bands was examined using SDS-PAGE, as described above. Electrophoresed 15% polyacrylamide gels were stained with 0.1% Coomassie Brilliant Blue dissolved in 50% methanol and 7% acetic acid for 30 min and decolorized with 7% acetic acid and 10% methanol. Nano-LC-MS/MS analysis of samples subjected to in-gel digestion with trypsin was performed using Nippon Proteomics, Sendai, Japan.

### 2.9 Expression level of MV-induced type I allergic reaction-related genes in RBL2H3 cells

Rat cell lines derived from basophilic leukemia (RBL)-2H3 cells were cultured in Dulbecco’s modified Eagle’s medium (Thermo Fisher Scientific, Waltham, MA, USA) supplemented with 20% fetal bovine serum (Thermo Fisher Scientific), Glutamax (Thermo Fisher Scientific), 100 units/mL penicillin, and 100 μg/mL streptomycin (Thermo Fisher Scientific) at 37°C under 5% CO_2_. RBL-2H3 cells were seeded in 24-well plates at 8.0 × 10^4^ cells/well, sensitized with 0.05 μg/mL anti-DNP IgE (Sigma-Aldrich) for 24 h at 37°C under 5% CO_2_, and washed twice with PBS. After washing, 180 μL of the releasing mixture (116.9 mM NaCl, 5.4 mM KCl, 0.8 mM Mg_2_SO_4_-7H_2_O, 5.6 mM Glucose, 25 mM HEPES, 2.0 mM CaCl_2_, and 1.0 mg/mL BSA; pH 7.7) and 20 μL of each isolated MV prepared with the mixed ratios of BHI broth:SAEW (1:0 [BHI broth:water solution, without SAEW treatment] and 1:2 [100 ng/mL protein concentration]) were added at 37°C and incubated for 10 min under 5% CO_2_. PBS was used as a control instead of MVs without SAEW treatment. After 10 min, 20 μL/well of DNP-BSA (15.2 ng/mL; Cosmo Bio, Tokyo, Japan) was added at a final concentration of 0.44 μg/mL and incubated for 30 min at 37°C under 5% CO_2_. The cells were observed under a microscope, and the reaction was stopped by ice cooling for 10 min. Cell lysis and reverse transcription reactions were performed using the CellAmp Direct TB Green RT-qPCR Kit (TaKaRa Bio Inc., Shiga, Japan) following the manufacturer’s instructions. The glyceraldehyde 3-phosphate dehydrogenase (GAPDH) gene was used as an internal standard to correct for mRNA levels between samples. Table 2 presents the primer sequences used.

**TABLE 2:** Sequences of primers used for analysis of type I allergic reaction-related genes.

Primer	Gene	Sequence (5′ to 3′)
HDC F	L-histidine decarboxylase	CTCCTTCACCTTTAACCCTTCCAAG
HDC R		ATCCATTGTCGTTTCCAGACTGTC
FcεR1α F	Fc epsilon receptor Ia	ACCGTGGATTAGAATACTTACAGGAG
FcεR1α R		CGTCAGCAGAAGATTGGAGCAG
IL-4 F	Interleukin-4	CGGTATCCACGGATGTAACGAC
IL-4 R		CCTCTACAGAGTTTCCTCAGTTCAC
TNF-α F	Tumor necrosis factor-α	AGATGTGGAACTGGCAGAGGAG
TNF-α R		TGGGCTACGGGCTTGTCAC
GAPDH F	Glyceraldehyde-3-phosphate dehydrogenase	GGTGATGCTGGTGCTGAGTATG
GAPDH R		GTCTTCTGAGTGGCAGTGATGG

### 2.10 Statistical analysis

All experimental results are presented as means ± standard deviation (SD). Two-group comparisons were performed using Student’s *t*-test. Analyses were performed using Microsoft Excel 2019 (Microsoft, Redmond, WA, USA). Two-tailed *P*-values of 0.05 were considered statistically significant.

## 3 Results

### 3.1 Effect of SAEW on *S. aureus* growth

**FIGURE 1:**
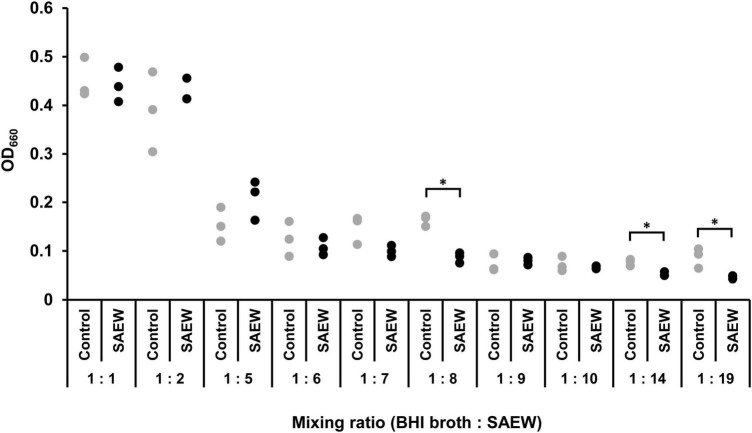
Effect of SAEW on the growth of *Staphylococcus aureus. S. aureus* growth was measured by optical density at 660 nm. The control used was sterile water instead of SAEW. *Indicates *p* < 0.05 compared to the control (*n* = 3).

The effect of SAEW on the change in the optical density of *S. aureus* C-29 was examined. Preliminary results showed that when *S. aureus* was inoculated in BHI broth with an initial bacterial count of 10^2^ CFU/mL (OD_660_ = 0.08) and incubated for 4 h with shaking, the bacterial count reached 2.1 × 10^4^ CFU/mL (OD_660_ = 0.14). From these results, it was determined that *S. aureus* grows at OD_660_ of 0.1 or higher. OD_660_ exceeded 0.1 when the mixing ratio was 1:1, 1:2, and 1:5, indicating the growth of *S. aureus* (Figure 1). Therefore, in subsequent experiments, mixing ratios of 1:1, 1:2, and 1:5 (BHI broth:SAEW) were used.

### 3.2 Effect of SAEW on virulence factor expression of *S. aureus*

**FIGURE 2:**
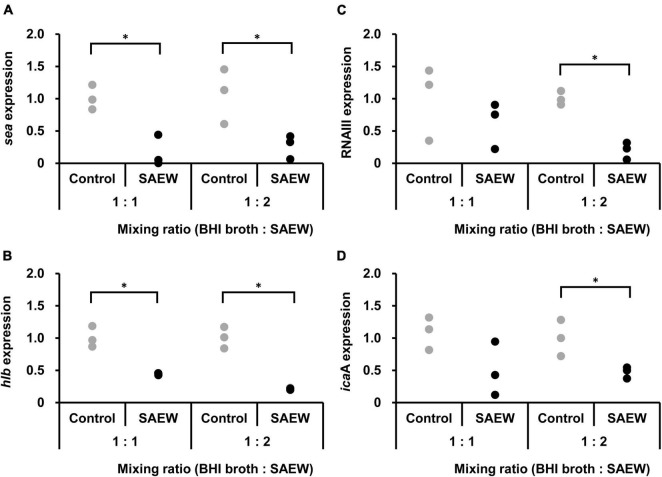
Effect of SAEW on virulence factor gene expression levels in *S. aureus*. **(A)** SEA gene (*sea*). **(B)** β-hemolysin gene (*hlb*). **(C)** RNAIII. **(D)** biofilm formation gene (*ica*A). *S. aureus* was added to the BHI broth:SAEW mixing ratios of 1:1 and 1:2 and incubated statically at 37°C for 6 h. The control used was sterile water instead of SAEW. The expression level of each gene in the control is shown as a relative value of 1. *Indicates *p* < 0.05 compared to the control (*n* = 3).

The effect of SAEW on the virulence gene expression of *S. aureus* C-29 was examined. Samples with mixing ratios of 1:1 and 1:2 were used because the amount of *S. aureus* in BHI broth:SAEW = 1:5 at 6 h of incubation was too low to extract total RNA. The results showed that *sea* and *hlb* expression levels were significantly reduced at mixing ratios of 1:1 and 1:2. The expression of *sea* was reduced 0.4- and 0.3-fold in the mixing ratios of 1:1 and 1:2 mixing ratios, respectively (Figure 2A). The expression of *hlb* was reduced 0.4- and 0.2-fold at 1:1 and 1:2 mixing ratios, respectively (Figure 2B). RNAIII level and *ica*A expression were significantly decreased in the mixing ratio of 1:2. RNAIII (Figure 2C) and *ica*A (Figure 2D) were reduced 0.2- and 0.5-times at 1:2 mixing ratio, respectively.

### 3.3 Effect of SAEW on the particle size of MVs from *S. aureus*

**FIGURE 3:**
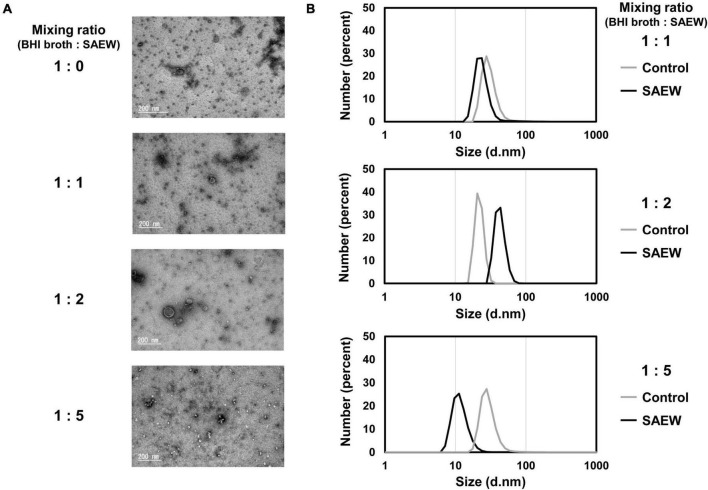
Effect of SAEW on the particle size of *S. aureus*-derived membrane vesicles (MVs). **(A)** TEM analysis of MVs. **(B)** Particle size distribution of MVs. The control (BHI broth:SAEW = 1:0) used was sterile water instead of SAEW.

The effect of SAEW on the particle size of *S. aureus*-derived MVs was measured. The presence of *S. aureus*-derived MVs was confirmed through TEM image analysis, with their sizes typically measuring approximately 50 nm (Figure 3A). Particle size measurements revealed that at a 1:1 mixing ratio, the MVs collected with sterile water and SEAW had average particle sizes of 30.9 ± 1.6 and 24.4 ± 2.6 nm, respectively (Figure 3B). At a mixing ratio of 1:2, the average particle sizes of the MVs collected with sterile water and SAEW were 22.1 ± 1.3 and 42.5 ± 2.1 nm, respectively (*P* < 0.05; Figure 3B), indicating that MVs with larger particle size were generated through SAEW treatment. At a mixing ratio of 1:5, the average particle sizes of MVs collected with sterile water and SAEW were 29.6 ± 2.0 and 11.7 ± 0.4 nm, respectively (*P* < 0.05; Figure 3B), indicating a smaller particle size of MVs.

### 3.4 Effect of SAEW on the amount of SEA in culture supernatant and MVs

**FIGURE 4:**
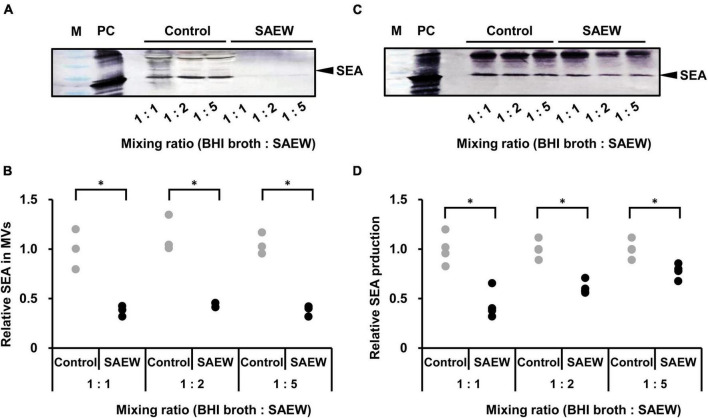
Effect of SAEW on staphylococcal enterotoxin A (SEA) content of culture supernatants and MVs of *S. aureus.* **(A)** SEA content of *S. aureus*-derived MVs analyzed by SDS-PAGE and visualized by western blotting analysis. **(B)** Measurement of SEA in *S. aureus*-derived MVs. **(C)** SEA production in the culture supernatant of *S. aureus* analyzed by SDS-PAGE and visualized by western blotting. **(D)** Measurement of SEA production in the culture supernatant of *S. aureus*. The control used was sterile water instead of SAEW. M: Pre-stained protein marker, and PC: positive control (SEA protein). Adjustments to the contrast and brightness were made using Adobe Photoshop to enable better visibility without altering the relationship between the SAEW images and the control images. The SEA in the control is shown as a relative value of 1. *Indicates *p* < 0.05 compared to the control (*n* = 3).

Evaluation of the effect of SAEW on SEA content in MVs revealed markedly reduced SEA content in MVs prepared at the mixed ratios of 1:1, 1:2, and 1:5 (Figures 4A, B). To determine whether SEA content in MVs was associated with decreased SEA production, the effect of SAEW on the SEA production of *S. aureus* was examined. SEA content in the culture supernatant of BHI broth:SAEW ratio of 1:1, 1:2, and 1:5 showed a decreasing trend, although it was not significant. To clarify that a decrease in the number of bacteria did not lead to a decrease in SEA production, the SEA protein concentration was normalized to the number of cells. The amount of SEA per cell was calculated by dividing the amount of SEA per culture medium by the number of bacteria. The results showed that the SEA production/cell was significantly reduced at mixed ratios of 1:1 and 1:2 (Figures 4C, D).

### 3.5 Effect of SAEW on the cargo protein composition of MVs

**FIGURE 5:**
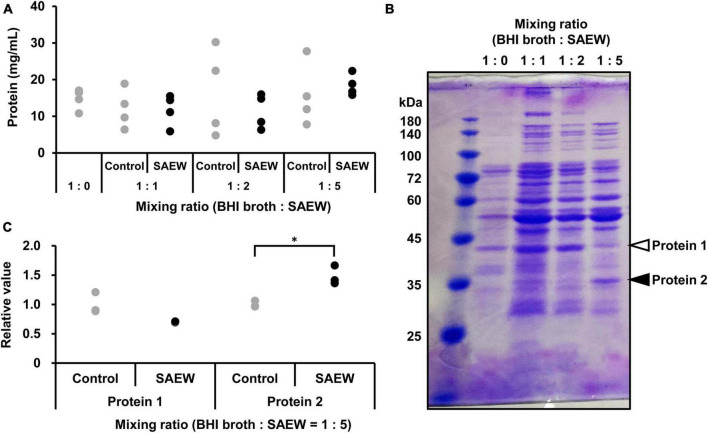
Effect of SAEW on cargo proteins of *S. aureus*-derived MVs. **(A)** Cargo protein concentration in *S. aureus*-derived MVs. **(B)** Cargo proteins in MVs obtained from supernatants after different SAEW mixing ratios. The control (BHI broth:SAEW = 1:0) used was sterile water instead of SAEW. Arrows (white): decreasing, arrows (filled): increasing. Bands indicated by arrows were subjected to nano-LC–MS/MS analysis. **(C)** Measurement of cargo proteins decreased or increased in *S. aureus*-derived MVs. Proteins 1 and 2 correspond to the bands in Figure 5B with the BHI broth:SAEW mixing ratio of 1:5. *p < 0.05 compared to the control (*n* = 3).

SDS-PAGE was performed to clarify the effect of SAEW on the cargo protein composition of MVs. The cargo protein concentrations in MVs were not significantly different (Figure 5A). Although the cargo protein composition of MVs prepared with the mixed ratios of 1:1 and 1:2 did not show notable changes in protein band patterns, the composition of MVs prepared with the mixed ratio of 1:5 noticeably changed (Figure 5B). SAEW decreased the number of proteins with a molecular weight of approximately 40 kDa (Figure 5B, protein 1) and increased the number of proteins with a molecular weight of approximately 30–35 kDa (Figure 5B, protein 2) in the MVs (Figure 5C). Accordingly, proteins in *S. aureus*-derived MVs whose expression was attenuated or enhanced by SAEW were identified using nano-LC–MS/MS analysis (Table 3). The results showed that the cargo proteins of MVs with reduced expression were pyruvate dehydrogenase E1 component subunits α and β, delta-aminolevulinic acid dehydratase (hemB), enolase, glyceraldehyde-3-phosphate dehydrogenase, SA1198 protein, and Fe/B_12_ periplasmic binding domain-containing protein. The cargo proteins of MVs with increased expression were periplasmic binding protein, lipoprotein, phage head protein, DNA-directed RNA polymerase subunit alpha, HPr kinase/phosphorylase, fructose-bisphosphate aldolase class 1, and lactate dehydrogenase.

**TABLE 3:** Identification of cargo protein in *Staphylococcus aureus*-derived MVs changed by SAEW.

Protein	Gene	MW	Accession	Mascot search accession No.
Pyruvate dehydrogenase E1 component subunit beta	*pdh*B	35,246	Q6GAC0	*WP_000068176.1*
Delta-aminolevulinic acid dehydratase	*hem*B	36,583	Q2FXR3	*WP_000667126.1*
Enolase	*eno*	47,117	O69174	*O69174.1*
Glyceraldehyde-3-phosphate dehydrogenase 1	*gap*A1	36,281	Q6GB58	*WP_000279414.1*
Pyruvate dehydrogenase E1 component subunit alpha	*pdh*A	41,383	P60089	*WP_000035320.1*
SA1198 protein		37,856	A0A0H3JLV4	*WP_000433354.1*
Fe/B_12_ periplasmic-binding domain-containing protein		37,854	Q2G071	*WP_000754443.1*
Periplasmic binding protein		34,025	A0A0E1XE76	*WP_000735034.1*
Periplasmic binding protein		36,687	A0A0E1XEH6	*WP_001214655.1*
Periplasmic binding protein, putative		36,744	Q2G1N4	*WP_001045111.1*
Lipoprotein		30,456	Q2G0V0	*WP_000825521.1*
Phage head protein, putative		36,771	Q2FX56	*WP_000438502.1*
DNA-directed RNA polymerase subunit alpha	*rpo*A	35,012	Q2FW32	*WP_000569649.1*
HPr kinase/phosphorylase	*hpr*K	34,482	Q2G045	*WP_000958224.1*
Periplasmic binding protein, putative		36,744	Q2G1N4	*WP_001045111.1*
Fructose-bisphosphate aldolase class 1	*fda*	32,913	Q6GDJ7	*WP_001031407.1*
D-lactate dehydrogenase		36,675		*WP_000161542.1*

### 3.6 Effects of MVs on genes related to type I allergic reactions

**FIGURE 6:**
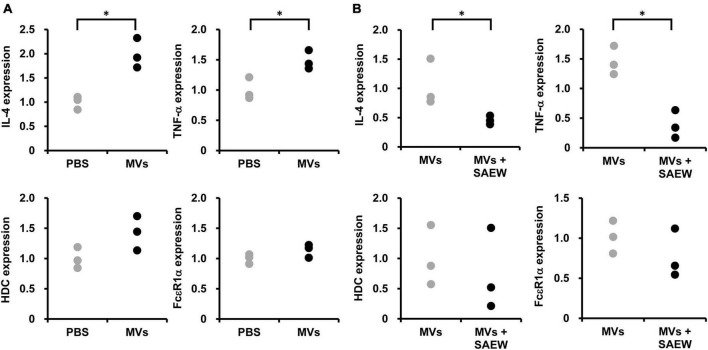
Effect of SAEW on *S. aureus*-derived MV-induced type I allergic reaction. *S. aureus*-derived MVs were exposed to IgE-sensitized RBL-2H3 cells. **(A)** Type I allergy-related gene expression induced by *S. aureus*-derived MVs. MVs obtained by culturing *S. aureus* in broth without SAEW. The control used was phosphate-buffered saline instead of MVs. **(B)** Effect of SAEW on *S. aureus*-derived MV-induced type I allergic reactions. MVs: MVs obtained by culturing *S. aureus* in broth without SAEW, MVs + SAEW: MVs obtained by culturing *S. aureus* in broth with SAEW. *Indicates *p* < 0.05 compared to the control (*n* = 3).

The effect of *S. aureus*-derived MVs on allergy-related gene expression in RBL-2H3 cells was examined. First, isolated MVs (without SAEW treatment) prepared in a mixed ratio of 1:0 were added to RBL-2H3 cells, and the mRNA expression levels of the allergy-related genes HDC, FcεR1α, IL-4, and TNF-α were examined. PBS was used as a control instead of MVs. The results showed that the addition of MVs (without SAEW treatment) markedly increased the expression levels of IL-4 and TNF-α compared with the control (PBS), whereas of the levels of HDC and FcεR1α were on an increasing trend (Figure 6A).

### 3.7 Effect of SAEW on MV-induced type I allergic reactions

The effect of SAEW on allergy-related gene expression in RBL-2H3 cells induced by *S. aureus*-derived MVs was examined. Comparison of the expression of HDC, FcεR1α, IL-4, and TNF-α mRNA in the MVs prepared with mixed ratios 1:0 (without SAEW treatment) and 1:2 (with SAEW treatment) revealed decreased levels of HDC, FcεR1α, TNF-α, and IL-4 in MVs (with SAEW treatment) (Figure 6B).

## 4 Discussion

Current treatment of AD relies mainly on a coping strategy, such as steroid-based immunosuppression, although there are other treatments such as skin care and environmental remediation. However, emerging therapies for AD are expected to leverage the antimicrobial action of natural products against *S. aureus* and their ability to inhibit the expression of virulence factors. SAEW (Kondo et al., 2006), known for its antimicrobial activity and safety, shows potential for AD treatment, but its use in this regard remains largely underexplored. Therefore, the effects of SAEW on *S. aureus* growth, virulence factor expression, and *S. aureus*-derived MV properties were examined in this study.

Slightly acidic electrolyzed water treatment at a mixing ratio of up to 1:5 favored *S. aureus* growth; however, the growth was inhibited by the SAEW ratio exceeding this value. The disinfection mechanism of SAEW relies on the oxidation of organic matter. Consequently, a higher level of coexisting organic matter reduces disinfection efficacy of SAEW (Rahman et al., 2016). In this study, the mixing of SAEW with BHI broth may have decreased the bactericidal effect. Mixing ratios of up to 1:5 were used in subsequent tests because higher mixing ratios, which inhibited *S. aureus* growth, may impede the effective evaluation of the expression of virulence factors. Evaluation of the effect of SAEW treatment on SEA production by *S. aureus* revealed that mixing ratios of 1:1, 1:2, and 1:5 markedly reduced SEA production per cell. Therefore, the effect of SAEW on SEA gene expression in *S. aureus* was examined, which revealed that mixing ratios of 1:1 and 1:2 resulted in markedly reduced expression levels. The SEA gene is carried by the temperate phage family (Betley and Mekalanos, 1985; Coleman et al., 1989), suggesting that SAEW may suppress SEA phage induction in *S. aureus*. In addition, the quorum sensing-regulated gene RNAIII and its downstream genes, *ica*A, associated with biofilm formation, and *hlb*, associated with hemolytic toxins, exhibited reduced expression levels. The expression of numerous virulence factors of *S. aureus* is regulated by the *agr* regulatory system through the QS mechanism. This system operates as a gene regulation mechanism that responds to bacterial density, and it functions by detecting a signal molecule known as an autoinducer peptide (AIP). It comprises four protein genes (*agr*BDCA) and regulatory RNAs called RNAII and RNAIII (small RNAs) that regulate the expression of many toxic factors such as *hld* (Jenul and Horswill, 2019). The RNAIII gene regulates the expression of a group of *agr* regulatory genes (Jenul and Horswill, 2019), suggesting that SAEW may inhibit the QS mechanism through the prevention of *S. aureus* from releasing AIP outside the bacteria and signaling it into the cell. *S. aureus* is thought to have a specific system to adapt to its environment and show virulence by infecting and surviving in the host during SAEW exposure.

As MVs contain bacteria’s cell membrane, the impact of SAEW on the cell membrane can change the release pattern and properties of these MVs. The evaluation of the effect of SAEW on the properties of *S. aureus*-derived MVs, specifically the particle size of MVs, revealed that the mixing ratio of 1:1 did not alter the particle size of MVs collected with the addition of SAEW, unlike MVs collected with sterile water. However, at a mixing ratio of 1:2, MVs collected with SAEW had larger particle sizes (22.1 ± 1.3–42.5 ± 2.1 nm) than those collected with sterile water, but contrasting observations were made at a mixing ratio of 1:5 (29.6 ± 2.0–11.7 ± 0.4 nm). It has been reported that the growth medium may affect the size distribution of MVs produced by *S. aureus* (Askarian et al., 2018). Hypochlorous acid is typically the most potent bactericidal chlorine species in SAEW, with a predominant percentage at pH 4–6. Despite being a polar molecule, this acid is small enough to permeate cell membranes, resulting in protein denaturation of the cell membrane/wall (Hazell and Stocker, 1993; Hazell et al., 1994; Hawkins and Davies, 1998, 1999) and oxidation of lipids (Spickett et al., 2000). Thus, hypochlorous acid in SAEW may have altered the cell membrane fluidity, resulting in the altered particle size of MVs. Usually, the size of *S. aureus*-derived MVs produced under *in vitro* conditions reaches approximately 100 nm, but the size of *S. aureus*-derived MVs in this study was smaller than that reported in other studies. The particle size of MVs of *S. aureus* MSSA476 cultured in BHI broth has been reported to be 24.4 ± 2.8 nm (Askarian et al., 2018). The reason for the small size of the MVs is unknown; however, the analysis of the TEM images and DSL results support that this is the correct size of MVs. As previously mentioned, coexisting molecules, ions, and other factors may affect the size of MVs and significantly influence their concentration, morphology, and ionic strength.

The evaluation of the effect of SAEW on the amount of toxin endosperm in MVs showed that the SEA content in MVs was markedly decreased following incubation with SAEW. SEA produced by *S. aureus* can induce food poisoning and systemic and local inflammatory reactions, leading to AD. Notably, SEA is highly expressed in human AD lesional tissues (Lee et al., 2013), suggesting that reducing the SEA content in MVs may lead to AD inhibition. In addition, an evaluation of the SEA content in the culture supernatant to examine the association between SEA content in MVs and decreased SEA production revealed that SEA content per cell was significantly decreased; however minor changes in SEA content in culture medium was observed. These results suggest that *S. aureus* selectively internalizes the SEA within MVs. The internalization of SEA into MVs may allow SEA to be delivered to host receptors at higher concentrations. The band above SEA in the western blot was inferred to be *Staphylococcus* protein A (*spa*), which non-specifically binds to IgG. The translation of *spa* mRNA is inhibited by RNAIII (Huntzinger et al., 2005). Therefore, a decrease in RNAIII levels in *S. aureus* was expected to increase the production of *Staphylococcus* protein A in the culture supernatant; however, in contrast, it tended to decrease compared with the control. The amount of RNAIII in the cell is not unlimited, especially in the middle of the exponential growth phase when the quorum sensing system is not yet fully activated (Le Scornet and Redder, 2019). This suggests that various target mRNAs must compete for binding to RNAIII. Many target mRNAs utilize the same binding sites on RNAIII, and how this competition affects the RNAIII-mediated regulation of virulence *in vivo* remains unclear.

Thus, *S. aureus* was thought to selectively internalize cargo necessary for survival within MVs to escape cell death by SAEW. SDS-PAGE was performed to examine alterations in the cargo proteins of *S. aureus*-derived MVs following SAEW exposure. The results showed that in MVs obtained in the mixture ratio of 1:5, the bands of proteins approximately 40 kDa decreased and those of approximately around 35 kDa increased. Therefore, the cargo proteins of these MVs with altered expression levels were identified using nano-LC–MS/MS analysis. Decreased expression proteins included pyruvate dehydrogenase E1 component subunit β, delta-aminolevulinic acid dehydratase; hemB, and Fe/B_12_ periplasmic binding protein. Pyruvate dehydrogenase E1 component subunit β is the most abundant protein in *S. aureus*-derived MVs (Gurung et al., 2011). HemB is an enzyme that oxidizes delta-aminolevulinic acid, the first precursor of heme and other tetrapyrrole compounds. HemB-deficient mutants have altered properties, such as retarded growth, decreased yellow pigmentation, reduced coagulase and hemolytic activities, and resistance to aminoglycosides (von Eiff et al., 1997). The Fe/B_12_ periplasmic binding protein is present in the periplasm between cell membranes and cell walls, and it incorporates Fe and vitamin B_12_ into the cytoplasm. Lysis of erythrocytes releases heme around them, allowing bacteria to assimilate free iron. Hemolytic toxins (hemolysins) are involved in iron acquisition, a limiting factor for the growth of pathogenic bacteria in the host body (Griffiths, 1991). The decreased expression of Fe/B_12_ periplasmic binding protein suggested that SAEW may have contributed to impeding *S. aureus* ability to assimilate free iron. Furthermore, proteins with increased expression included periplasmic binding protein, phage head protein, and lactate dehydrogenase. Periplasmic binding proteins are located in the periplasm between the cell membrane and cell wall. The production of MVs is as a surface stress response system that releases heterologous proteins in the periplasm (McBroom and Kuehn, 2007), implying that SAEW may have induced stress in *S. aureus* and increased the release of proteins from the periplasm into the MVs. Lactate dehydrogenase can be induced when *S. aureus* is under stress conditions and glutathione is present in cells (Christmas et al., 2019). This suggests that *S. aureus* was exposed to stress by SAEW, resulting in increased levels of lactate dehydrogenase.

Allergic symptoms are triggered by a complex interplay of lymphocytes (T cells and B cells); mast cells containing histamine and prostaglandins, which are chemical messengers that induce allergic symptoms; and cytokines produced by these cells. Mast cells, in particular, contain L-histidine decarboxylase (HDC), an enzyme that synthesizes histamine from histidine, and express FcεRI, a receptor for IgE antibodies, on the cell surface. It also contains cytokines, such as IL-4 and TNF-α that are involved in the exacerbation of allergic symptoms and play an important role in allergic reactions (Kawakami and Kasakura, 2019). Previous studies have shown that *S. aureus*-derived MVs enhance the release of β-hexosaminidase, an indicator of degranulation induction in RBL-2H3 cells (Yamanashi et al., 2022). Furthermore, the protein composition in the released MVs was different when *S. aureus* was cultured in the presence of SAEW, suggesting that changes in the composition of MVs may affect allergic responses. Therefore, the effects of SAEW on the expression of allergy-related genes were examined using RBL-2H3 cells induced by *S. aureus*-derived MVs. The results showed that the expression of HDC and FcεR1α tended to decrease in MVs (MVs + SAEW) obtained by culturing *S. aureus* in the presence of SAEW. In addition, the expression levels of TNF-α and IL-4 decreased notably in the MVs + SAEW. These results suggest that SAEW may reduce the internalization of substances involved in the exacerbation of allergic symptoms in *S. aureus*-derived MVs. *S. aureus*-derived MVs are rich in virulence factors within their lipid bilayers; MVs are thought to more stably store and effectively transport these molecules (Hong et al., 2011). SAEW decreased SEA content in MVs from *S. aureus*. Therefore, the effect of SEA on allergy-related gene expression in RBL-2H3 cells was examined. In contrast to MVs, SEA did not increase the expression of TNF or IL-4. The decrease in allergy-related gene expression was thought to be related to SAEW-induced changes in components other than SEA in MVs. *S. aureus* expresses several short amphipathic peptides called phenol soluble modulins (PSMs)—a group of molecules containing δ-toxins encoded within RNAIII, which is the QS regulatory gene of *S. aureus* (Peschel and Otto, 2013). δ-toxin can cause degranulation, specifically without lysing mast cells derived from fetal skin or bone marrow (Nakamura et al., 2013). Although δ-toxin was not analyzed in this study, as SAEW significantly reduced RNAIII expression in *S. aureus*, it may have a reducing effect on δ-toxin. In this study, we demonstrated that SAEW suppressed *S. aureus* growth and MV-induced virulence factor expression and inflammation. Further studies are expected to identify the factors responsible for altering the properties of *S. aureus*-derived MVs involved in AD pathogenesis.

**FIGURE 7:**
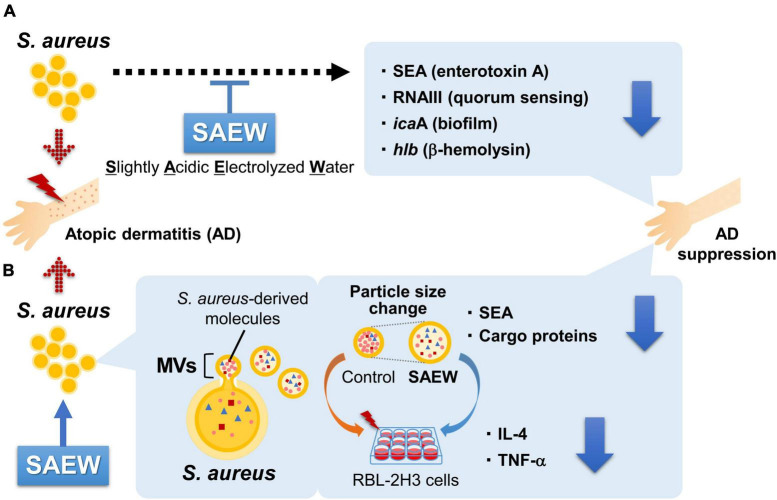
Effects of SAEW on the virulence factors of *S. aureus* and MVs. **(A)** *S. aureus* cultured with SAEW reduced SEA production and decreased the expression of virulence factor-related genes. **(B)** MVs from SAEW-supplemented broth displayed altered particle sizes, and markedly reduced contents of SEA and cargo proteins. Allergy-related gene expression in RBL-2H3 cells was substantially reduced in MVs obtained from the SAEW-treated broth.

In this study, a series of culture experiments was performed using MV obtained from *S. aureus* exposed to SAEW at various ratios (1, 2, and 5) to broth, followed by the comprehensive analysis of changes in inflammatory markers, gene expression profiles, and protein cargo. *S. aureus* cultured with SAEW at ratios of 1, 2, and 5 per broth exhibited reduced SEA production and expression of virulence factor-related genes (Figure 7A). Our study clarified that SAEW affects the inclusion of SEA and cargo proteins in *S. aureus*-derived MVs. The study also clarified that SAEW inhibited allergy-related gene expression by changes in the inclusion components of MVs (Figure 7B). Overall, this study offers new insights into the modulation of *S. aureus*-derived MV-induced inflammation, which is central to AD pathogenesis. It also highlights the potential of SAEW as a promising therapeutic agent in managing AD-associated inflammation. It is unclear whether SAEW is beneficial, even for brief exposures. The hypochlorous acid in SAEW rapidly decomposes upon contact with organic matter, passing oxygen to the organic matter and losing its activity within a short period of time (Rahman et al., 2016). However, it is expected that the concentrations of hypochlorous acid would allow it to remain effective to some extent. Furthermore, in this study, the effect of SAEW on SEA-producing *S. aureus* was analyzed, focusing on its application to AD. SEA is not the staphylococcal superantigen that is most strongly associated with AD, and most reports have shown the influence of SEB on the pathogenesis and course of this disease (Merriman et al., 2016). However, as reported in a recent meta-analysis, the prevalence of IgE in SEA (33%) was as high as in SEB (35%) in patients with AD compared with controls (Blicharz et al., 2022). To support the clinical application of study results, the effect of SAEW on SE-producing strains other than SEA and their MVs should be investigated, and further *in vivo* studies should be conducted.

## Data availability statement

The original contributions presented in the study are included in the article/supplementary material, further inquiries can be directed to the corresponding author.

## Author contributions

YS: Data curation, Formal analysis, Supervision, Visualization, Writing – original draft. YO: Formal analysis, Visualization, Writing – review and editing. MT: Formal analysis, Visualization, Writing – review and editing. YY: Formal analysis, Visualization, Writing – review and editing. AO: Formal analysis, Writing - review and editing. MO: Formal analysis, Writing - review and editing. MK: Formal analysis, Writing - review and editing. KS: Formal analysis, Writing – review and editing. SM: Data curation, Supervision, Writing – review and editing.
